# Chaenomelin, a New Phenolic Glycoside, and Anti-*Helicobacter pylori* Phenolic Compounds from the Leaves of *Salix chaenomeloides*

**DOI:** 10.3390/plants13050701

**Published:** 2024-02-29

**Authors:** Kyung Ah Kim, Dong-Min Kang, Yoon-Joo Ko, Moon-Jin Ra, Sang-Mi Jung, Jeong-Nam Yu, Mi-Jeong Ahn, Ki Hyun Kim

**Affiliations:** 1 School of Pharmacy, Sungkyunkwan University, Suwon 16419, Republic of Korea; ruddk5480@naver.com; 2 College of Pharmacy and Research Institute of Pharmaceutical Sciences, Gyeongsang National University, Jinju 52828, Republic of Korea; kdm7105@gnu.ac.kr (D.-M.K.); amj5812@gnu.ac.kr (M.-J.A.); 3 Laboratory of Nuclear Magnetic Resonance, National Center for Inter-University Research Facilities (NCIRF), Seoul National University, Gwanak-gu, Seoul 08826, Republic of Korea; yjko@snu.ac.kr; 4 Hongcheon Institute of Medicinal Herb, Hongcheon-gun 25142, Republic of Korea; ramj90@himh.re.kr (M.-J.R.); sgmo77@naver.com (S.-M.J.); 5 Nakdonggang National Institute of Biological Resources, Sangju 37242, Republic of Korea; susia000@nnibr.re.kr

**Keywords:** *Salix chaenomeloides*, phenolic glycoside, chaenomelin, *Helicobacter pylori*

## Abstract

*Salix chaenomeloides* Kimura, commonly known as pussy willow, is a deciduous shrub and tree belonging to the Salicaceae family. The genus *Salix* spp. has been known as a healing herb for the treatment of fever, inflammation, and pain relief. The current study aimed to investigate the potential bioactive natural products from *S. chaenomeloides* leaves and evaluate their antibacterial activity against *Helicobacter pylori*. A phytochemical investigation of the ethanol (EtOH) extract of *S. chaenomeloides* leaves led to the isolation of 13 phenolic compounds (**1**–**13**) from the ethyl acetate (EtOAc) fraction, which showed antibacterial activity against *H. pylori* strain 51. The chemical structure of a new phenolic glycoside, chaenomelin (**1**), was established by a detailed analysis of 1D and 2D (^1^H-^1^H correlation spectroscopy (COSY), heteronuclear single-quantum coherence (HSQC), and heteronuclear multiple-bond correlation (HMBC)) nuclear magnetic resonance (NMR), high-resolution electrospray ionization mass spectroscopy (HR-ESIMS), and chemical reactions. The other known compounds were identified as 5-*O*-*trans-p-*coumaroyl quinic acid methyl ester (**2**), tremulacin (**3**), citrusin C (**4**), benzyl 3-*O*-*β*-d-glucopyranosyl-7-hydroxybenzoate (**5**), tremuloidin (**6**), 1-[*O*-*β*-d-glucopyranosyl(1→2)-*β*-d-glucopyranosyl]oxy-2-phenol (**7**), arbutin cinnamate (**8**), tremulacinol (**9**), catechol (**10**), 4-hydroxybenzaldehyde (**11**), kaempferol 3-rutinoside (**12**), and narcissin (**13**), based on the comparison of their NMR spectra with the reported data and liquid chromatography/mass spectrometry (LC/MS) analysis. The isolated compounds were evaluated for antibacterial activity against *H. pylori* strain 51. Among the isolates, 1-[*O*-*β*-d-glucopyranosyl(1→2)-*β*-d-glucopyranosyl]oxy-2-phenol (**7**) and arbutin cinnamate (**8**) exhibited antibacterial activity against *H. pylori* strain 51, with inhibitions of 31.4% and 33.9%, respectively, at a final concentration of 100 μM. These results were comparable to that of quercetin (38.4% inhibition), which served as a positive control. Generally, these findings highlight the potential of the active compounds **7** and **8** as antibacterial agents against *H. pylori*.

## 1. Introduction

*Salix chaenomeloides* Kimura (=*S. glandulosa* Seemen), commonly known as Korean pussy willow in South Korea, is a deciduous shrub and tree belonging to the Salicaceae family. It is native to eastern Asia, including regions of Korea, China, Japan, and Russia. This plant species is known for its ornamental qualities, ecological importance, and potential as a source of bioactive compounds with diverse pharmacological properties [1,2]. For thousands of years, the genus *Salix* spp. has been recognized as a healing herb used for the treatment of fever, inflammation, and pain relief, owing to the presence of anti-inflammatory compounds in their bark, including salicylic acid, a widely recognized natural precursor to aspirin [1,2]. In a related context, previous pharmacological investigations involving the extracts of *Salix* spp. have revealed a range of therapeutic benefits, including antioxidant [3], anti-tumor [4], anti-inflammatory [5], and anti-obesity effects [6]. Phytochemical investigations of the genus *Salix* have led to reports on phenolic compounds, flavonoids, terpenoids, and lignans, known to play crucial roles in plant defense mechanisms and interactions with the environment. These compounds have been associated with various biological activities [7,8,9,10,11], such as cytotoxic, neuroprotective, and antiplasmodial effects. Despite their beneficial effects, only a limited number of studies on biological and phytochemical investigations of *S. chaenomeloides* have been reported. A previous phytochemical study on *S. chaenomeloides* documented the existence of flavonoids, phenolic glycosides, and salicin derivatives, and previous pharmacological studies have revealed that these compounds in *S. chaenomeloides* exhibit anti-inflammatory and neuroprotective properties against LPS-induced neuronal death [12,13]. Consequently, it was deemed necessary to conduct further investigations into the bioactive natural products from *S. chaenomeloides*.

The exploration of novel and bioactive natural products stands as a pivotal journey in the realm of scientific discovery and healthcare innovation [14]. Natural products, with their chemical diversity, offer the promise of yielding innovative therapeutics [15]. Particularly, historical evidence attests to the effectiveness of natural products as antibiotics [16]. Natural products also hold promise in addressing the mounting burden of mental health and neurological disorders, providing novel, side-effect-minimized treatments [16,17]. One of the hallmarks of natural products is their high specificity and lower toxicity compared to synthetic counterparts [18]. This characteristic diminishes the risk of adverse side effects, underpinning a safer platform for drug development and the prospect of more effective and well-tolerated treatments. Thus, natural products serve as an invaluable wellspring of inspiration for medicinal chemists and pharmaceutical researchers.

In our ongoing effort to advance our exploration of novel and bioactive metabolites derived from intriguing natural sources [19,20,21,22,23], we initiated a study with the aim of discovering potential bioactive compounds from the ethanol (EtOH) extract of *S. chaenomeloides* leaves, given that the EtOH extract demonstrated antibacterial activity against *Helicobacter pylori* strain 51. In the present study, the EtOH extract of *S. chaenomeloides* leaves underwent solvent partitioning, and the solvent-partitioned fractions were subjected to antibacterial activity tests in our bioactivity screening, where we observed that the ethyl acetate (EtOAc) fraction exhibited antibacterial activity against *H. pylori* strain 51. Comprehensive chemical analysis of the EtOAc fraction through repeated column chromatography and semi-preparative HPLC led to the isolation and identification of thirteen phenolic compounds (**1**–**13**), which included a previously unreported phenolic glycoside (**1**). The structure of the new compound (**1**) was established through a detailed analysis involving 1D and 2D nuclear magnetic resonance (NMR) spectroscopic techniques, including ^1^H-^1^H correlation spectroscopy (COSY), heteronuclear single-quantum coherence (HSQC), and heteronuclear multiple-bond correlation (HMBC) as well as high-resolution electrospray ionization mass spectroscopy (HR-ESIMS) and chemical reactions. Furthermore, our study investigated the potential bioactivity of these compounds, with a particular focus on their antibacterial activity against *H. pylori*.

## 2. Results and Discussion

### 2.1. Isolation and Structural Elucidation of Compounds ***1***–***13***

**Figure 1:**
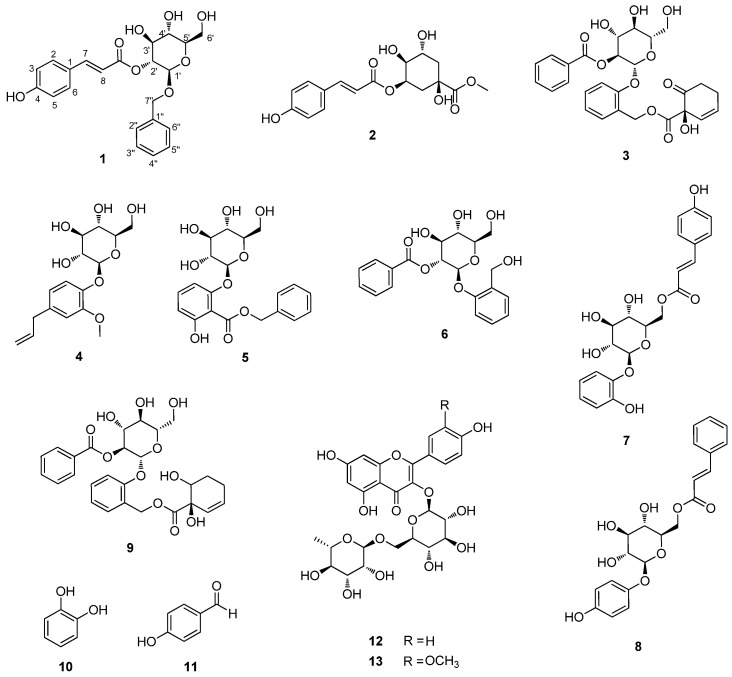
Chemical structures of compounds **1**–**13**.

The EtOH extract of *S. chaenomeloides* leaves, exhibiting antibacterial activity against *H. pylori* strain 51, underwent solvent partitioning using four organic solvents: hexane, CH_2_Cl_2_, EtOAc, and BuOH. Liquid chromatography/mass spectrometry (LC/MS) analysis of the fractions revealed the presence of several peaks representing phenolic compounds in the EtOAc-soluble fraction. These compounds were identified as the main components through a comparison with our in-house UV library in LC/MS. In our bioactivity screening test, we confirmed the EtOAc-soluble fraction as the antibacterial fraction against *H. pylori* strain 51 and investigated the active EtOAc fraction using continuous column chromatography and semi-preparative HPLC, guided by LC/MS analysis. This extensive analysis resulted in the isolation of thirteen compounds (**1**–**13**), as illustrated in Figure 1.

Compound **1** was isolated as a white amorphous powder. The molecular formula of **1** was established as C_22_H_24_O_8_, determined by negative-ion HR-ESIMS (Figure S1), which revealed an [M–H]^−^ ion peak at *m*/*z* 415.1414 (calcd. for C_22_H_23_O_8_, 415.1398). The ^1^H NMR data (Table 1) of **1** (Figure S3), assigned by the aid of the heteronuclear single-quantum correlation (HSQC) experiment (Figure S5), displayed the presence of characteristic signals of a 1,4-disubstituted aromatic ring at *δ*_H_ 7.47 (2H, d, *J* = 8.5 Hz, H-2, 6) and 6.83 (2H, d, *J* = 8.5 Hz, H-3, 5), a *trans*-double bond at *δ*_H_ 7.61 (1H, d, *J* = 16.0 Hz, H-7) and 6.33 (1H, d, *J* = 16.0 Hz, H-8), an anomeric proton at *δ*_H_ 4.56 (1H, d, *J* = 8.0 Hz, H-1′), a 1-monosubstituted aromatic ring at *δ*_H_ 7.27 (2H, d, *J* = 7.0 Hz, H-2″, 6″) and 7.22 (3H, m, H-3″, 4″, 5″) and oxygenated methylene at *δ*_H_ 4.87 (1H, d, *J* = 12.5 Hz, H-7″a) and 4.64 (1H, d, *J* = 12.5 Hz, H-7″b). The ^13^C NMR data (Table 1) revealed 22 carbon resonances, including four non-protonated carbons [*δ*_C_ 168.6, 161.5, 139.2, and 127.5], nine protonated aromatic carbons [*δ*_C_ 131.3 (×2), 129.3 (×2), 129.1, 129.0 (×2), 116.9 (×2)], a *trans-*double bond [*δ*_C_ 146.9 and 115.4], oxygenated methylene [*δ*_C_ 71.6], and sugar moiety signals [*δ*_C_ 101.4, 78.3, 76.2, 75.2, 71.8, and 62.8]. The ^1^H and ^13^C NMR spectroscopic features suggested that compound **1** may be a phenolic glycoside that is similar to 1-[*O*-*β*-d-glucopyranosyl(1→2)-*β*-d-glucopyranosyl]oxy-2-phenol (**7**) [24] and arbutin cinnamate (**8**) [25].

**Figure 2:**
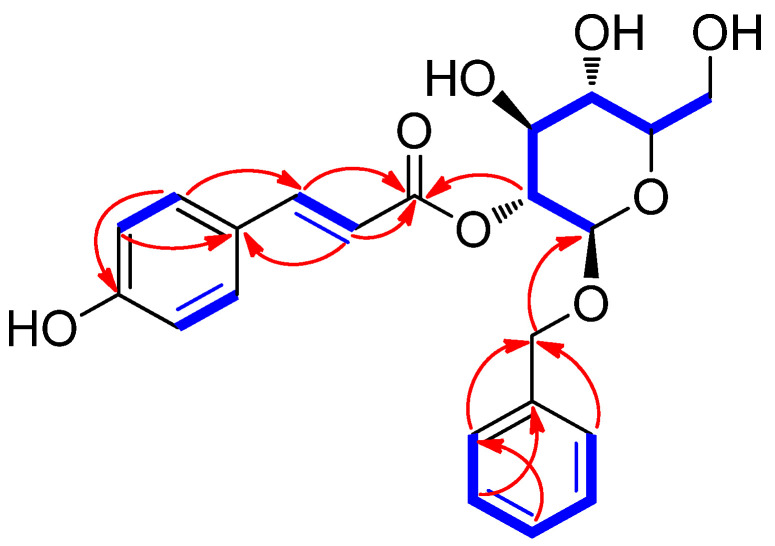
1H-1H COSY (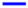) and HMBC (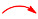) correlations for compound 1.

The planar structure of **1** was determined by the analysis of 2D NMR (COSY and HMBC) spectroscopic data (Figures S4 and S6). The ^1^H-^1^H COSY correlations of H-2/H-3, H-5/H-6, H-7/H-8, and H-2″/H-3″/H-4″/H-5″/H-6″ were observed. The HMBC correlations from H-2 (H-6) to C-4, from H-2 (H-6) to C-7, from H-8 to C-1, and from H-3 (H-5) to C-1, indicated the presence of the 1,4-disubstituted aromatic ring and *trans*-double bond (Figure 2). The key HMBC correlations of H-7/C-9 and H-8/C-9 and the HMBC correlations of H-2′/C-9 confirmed the connection of glycoside at H-2′ with an ester moiety (Figure 2). Furthermore, the HMBC correlations from H-7″ to C-1′ and H-2″ (H-6″) to C-7″ revealed that benzyl alcohol was connected to C-1′ (Figure 2). To confirm the sugar unit, β-glucosidase (1 mg, almonds, Sigma-Aldrich) was utilized for the enzymatic hydrolysis of **1**, but it initially failed to hydrolyze the sugar unit [12]. Then, the subsequent acid hydrolysis of **1** gave D-glucopyranose, which was verified by LC/MS analysis [26]. Finally, the coupling constant of the anomeric proton at *δ*_H_ 4.56 (1H, d, *J* = 8.0 Hz, H-1′) confirmed the β-d-glucopyranose. Based on the above data, the chemical structure of **1** was determined, as shown in Figure 1, and named chaenomelin.

We identified the known compounds as 5-*O*-*trans-p-*coumaroyl quinic acid methyl ester (**2**) [27], tremulacin (**3**) [28], citrusin C (**4**) [29], benzyl 3-*O*-*β*-d-glucopyranosyl-7-hydroxybenzoate (**5**) [30], tremuloidin (**6**) [31], 1-[*O*-*β*-d-glucopyranosyl(1→2)-*β*-d-glucopyranosyl]oxy-2-phenol (**7**) [24], arbutin cinnamate (**8**) [25], tremulacinol (**9**) [28], catechol (**10**) [32], 4-hydroxybenzaldehyde (**11**) [33], kaempferol 3-rutinoside (**12**) [34], and narcissin (**13**) [35] based on the comparison of their NMR spectra with the reported data and LC/MS analysis.

### 2.2. Evaluation of Antibacterial Activity of the Isolated Compounds against Helicobacter pylori

Natural products have historically yielded some of the most effective antimicrobial agents, and the discovery of new antimicrobial compounds from nature offers hope in the battle against drug-resistant superbugs. These new antibiotics can serve as essential tools to treat infections that were once manageable but have now become perilous [36,37]. *Helicobacter pylori* represents a significant global public health concern, affecting approximately 50% of the world’s population [38]. The eradication of *H. pylori* is crucial for the treatment of gastric and duodenal ulcers, as well as gastric cancer [39], given its known association with these pathologies. In previous studies, extracts of *Salix* spp. have demonstrated antimicrobial activity against Gram-positive bacteria (*Staphylococcus aureus* and *Bacillus subtilis*) and Gram-negative bacteria (*Escherichia coli*, *Klebsiella pneumoniae*, *Salmonella enterica*, and *Pseudomonas aeruginosa*) [4,40,41]. However, despite possessing such potent antimicrobial activity, there is currently no research on *Salix* spp. antimicrobial activity activity against *H. pylori*. Consequently, we proceeded to assess the antimicrobial activity of *S. chaenomeloides* against *H. pylori*.

In our bioactivity screening test aimed at discovering potential bioactive natural resources, the EtOH extract of *S. chaenomeloides* leaves exhibited antibacterial activity against *H. pylori* strain 51 with a 12.7% inhibition at a concentration of 100 μg/mL. Among the solvent-partitioning fractions derived from the EtOH extract, we identified the EtOAc fraction as the antibacterial fraction against *H. pylori* strain 51 with a 22.4% inhibition at a concentration of 100 μg/mL. Subsequently, we isolated compounds **1**–**13** from the active EtOAc fraction and assessed them for their antibacterial activity. While it has been reported that phenolic glycosides exhibit antimicrobial activity against bacteria like *S. aureus*, *E. faecalis*, and *B. cereus* [42,43], there is limited research on the antimicrobial activity of phenolic glycosides against *H. pylori*. Therefore, we conducted antimicrobial tests on the isolated compounds to explore their potential antimicrobial effects against *H. pylori*. Among the compounds tested, compounds **7** and **8** [1-[*O*-*β*-d-glucopyranosyl(1→2)-*β*-d-glucopyranosyl]oxy-2-phenol (**7**) and arbutin cinnamate (**8**)] demonstrated antibacterial activity against *H. pylori* strain 51, with inhibitions of 31.4% and 33.9%, respectively, at a final concentration of 100 μM (Table 2). These results were comparable to that of quercetin (38.4% inhibition), which served as a positive control [44]. The other compounds either exhibited weak activity or failed to show anti-*H. pylori* effects.

On the other hand, it is necessary to analyze the results from our antimicrobial activity test of compounds **1**–**13**, comparing them to those from previous studies using the same or similar compounds. According to a recent study [45], several quinic acid derivatives were evaluated for their antibacterial properties against five bacterial strains. These strains encompassed both Gram-positive, including *S. aureus* and *Bacillus thuringiensis*, and Gram-negative bacteria, such as *E. coli*, *S. enterica*, and *Shigella dysenteria*. The experimental findings unveiled that the tested compounds, 5-*O*-*trans*-*O*-coumaroylquinic acid methyl ester, chlorogenic acid methyl ester, macranthoin F, and macranthoin G, exhibited notable activity against all five bacterial strains under investigation. Notably, macranthoin F and macranthoin G demonstrated exceptional in vitro bacteriostatic activity against *S. enterica*, with minimal inhibitory concentration (MIC) values of 7.4 and 14.7 μM, respectively. These MIC values are in close proximity to the positive-control compound kanamycin, which exhibited an MIC of 3.4 μM against *S. enterica*. However, one of the quinic acid derivatives, 5-*O*-*trans-p-*coumaroyl quinic acid methyl ester (**2**) isolated from *S. chaenomeloides* leaves in this study, did not show antimicrobial activity against *H. pylori* (Table 2). Notably, the new compound, chaenomelin (**1**), exhibited weak antibacterial activity against *H. pylori*, with an inhibition of 21.6%, which did not compare favorably to that of quercetin (38.4% inhibition). A literature survey on the antimicrobial activity of compounds with a structure similar to **1** revealed that samioside from *Phlomis samia* demonstrated weak antimicrobial activity against both Gram-positive (*S. aureus* and *S. epidermidis*) and Gram-negative bacteria (*Enterobacter cloacae*, *E. coli*, *K. pneumoniae*, and *P. aeruginosa*) [46]. Verbascoside and pedicularioside G, isolated from *Pogostemon cablin*, displayed weak antibacterial activity against two Gram-positive bacteria, *B. subtilis* and *S. aureus* [47]. Additionally, lianqiaoxinoside B from *Forsythia suspensa* exhibited weak antimicrobial activities against four common bacteria as follows: *Bacterium vulgare*, *Aeruginosus bacillus*, *Micrococcus pneumoniae*, and *B. dysenteriae*, when compared to cefalexin as the positive control [48], and isoforsythiaside and forsythiaside, isolated from *F*. *suspensa*, showed weak antimicrobial activities against *E. coli* [49]. Taken together, we can conclude that the results of previous studies demonstrated weak antimicrobial activity of phenolic compounds with a structure similar to compound **1** against various bacteria, which aligns with the findings of our antimicrobial activity test. To the best of our knowledge, this marks the first result indicating anti-*Helicobacter pylori* activity in phenolic compounds with a structure similar to **1**, although it showed weak activity. Further structural modifications may be necessary to demonstrate more potent antimicrobial activity for compound **1**.

## 3. Materials and Methods

### 3.1. Equipment Used for Analyses

The equipment and devices used in the analyses and experiments are listed in Table 3.

### 3.2. Plant Material

*S. chaenomeloides* leaves were collected in May 2022 from Goesan-gun, Chungcheongbuk-do, Republic of Korea. A voucher specimen (HIMH-2213) was identified and authenticated by Dr. Hye-Ryen Na at the Northeastern Asia Biodiversity Institute, located in Seoul 05677, Republic of Korea. The collected material has been deposited in the herbarium of the Nakdonggang National Institute of Biological Resources in Sangju, Republic of Korea.

### 3.3. Extraction and Isolation

The dried leaves (1.1 kg) of *S. chaenomeloides* were extracted with 80% ethanol (10 L) under reflux. The extract was condensed using a rotary evaporator to obtain a brown crude extract (80.5 g). The crude extract (30 g) was dissolved in distilled water (700 mL) and partitioned with hexane, dichloromethane (CH_2_Cl_2_), ethyl acetate (EtOAc), and *n*-butanol (*n*-BuOH). Four layers with increasing polarity, including the hexane, CH_2_Cl_2_, EtOAc, and *n*-BuOH soluble fractions, were obtained as 1.5, 5.8, 4.1, and 6.1 g, respectively. Based on the LC-MS analysis of each fraction derived from solvent partitioning, it was confirmed that the EtOAc-soluble fraction (4.1 g) contains phenolic glycosides as major compounds. The EtOA-soluble fraction was subjected to reversed-phase silica open-column chromatography with a gradient solvent system of MeOH/H_2_O (20~100% MeOH) to obtain five fractions (SA–SE). Fraction SA (2.9 g) was subjected to normal-phase silica open-column chromatography with a gradient solvent system of CH_2_Cl_2_/MeOH (15:1, CH_2_Cl_2_/MeOH to 100% MeOH) to yield nine fractions (SA1-SA9). Subfraction SA1 (23.1 mg) was subjected to semi-preparative reversed-phase HPLC with a 22% MeCN/H_2_O isocratic solvent system to isolate compounds **10** (2.8 mg, *t*_R_ = 15.7 min) and **11** (1.0 mg, *t*_R_ = 19.5 min). Subfraction SA4 (75.0 mg) was subjected to semi-preparative reversed-phase HPLC with a 36% MeCN/H_2_O isocratic solvent system to isolate compounds **2** (1.0 mg, *t*_R_ = 8.6 min) and **3** (21.0 mg, *t*_R_ = 20.8 min). Subfraction SA5 (170.3 mg) was fractioned by preparative reversed-phase HPLC with a gradient solvent system of MeOH/H_2_O (from 35% to 80% MeOH) to obtain five fractions (SA51–SA55). Subfraction SA54 (54.1 mg) was subjected to semi-preparative reversed-phase HPLC with a 24% MeCN/H_2_O isocratic solvent system to isolate compounds **4** (1.0 mg, *t*_R_ = 26.5 min), **5** (0.7 mg, *t*_R_ = 29.8 min), and **6** (4.5 mg, *t*_R_ = 38.2 min). Subfraction SA55 (40.1 mg) was subjected to semi-preparative reversed-phase HPLC with a 27% MeCN/H_2_O isocratic solvent system to isolate compounds **7** (0.6 mg, *t*_R_ = 29.0 min), **1** (2.2 mg, *t*_R_ = 38.0 min), **8** (0.8 mg, *t*_R_ = 41.5 min), and **9** (2.1 mg, *t*_R_ = 45.7 min). Subfraction SA9 was subjected to Sephadex-LH20 column chromatography to obtain three fractions (SA91–SA93). Subfraction SA93 (45.0 mg) was subjected to semi-preparative reversed-phase HPLC with a 19% MeCN/H_2_O isocratic solvent system to isolate compounds **12** (3.5 mg, *t*_R_ = 33.4 min) and **13** (3.4 mg, *t*_R_ = 36.3 min).

Chaenomelin (**1**)

White amorphous powder; [$\alpha_{D}^{25}$]-43.0 (*c* 0.04, MeOH); UV (MeOH): λ_max_ (log *ε*) 235 (2.5), 312 (3.2) nm (Figure S2); IR (neat): *ν*_max_ = 3390, 2940, 1696, 1600, 1156, and 1084 cm^−1^; and ^1^H (850 MHz) and ^13^C (212.5 MHz) NMR data, see Table 1. HR-ESIMS (negative-ion mode) *m/z* 415.1414 [M−H]^−^ (calcd. for C_22_H_23_O_8_, 415.1398).

### 3.4. Acid Hydrolysis of ***1***

Compound **1** (1.0 mg) was hydrolyzed in the presence of 1 N HCl (1.0 mL) at 90 °C for 2 h, and EtOAc was used for the extraction. The aqueous layer was neutralized with repeated evaporation under a vacuum evaporator and dissolved in anhydrous pyridine (0.5 mL) with the addition of L-cysteine methyl ester hydrochloride (2.0 mg). *O*-tolyl isothiocyanate (50 μL) was added to the reaction mixture and heated at 60 °C for an additional 1 h [21]. The reaction product was evaporated under a vacuum evaporator and dissolved in MeOH. Next, the dissolved reaction product was directly analyzed by LC/MS [MeOH/H_2_O, 1:9 → 6:4 gradient system (0–30 min), 100% MeOH (31–41 min), 0% MeOH (42–52 min); flow rate of 0.3 mL/min] using an analytical Kinetex C18 100 Å column (100 mm × 2.1 mm i.d., 5 μm). The sugar moiety from **1** was identified as D-glucopyranose based on the LC/MS analysis.

### 3.5. Anti-Helicobacter pylori Activity

A clinical strain, *H. pylori* 51, isolated from a Korean patient with a duodenal ulcer (HPKTCC B0006), was a general gift from the *H. pylori* Korean Type Culture Collection, School of Medicine, Gyeongsang National University, Korea. The strain was grown and maintained in Brucella broth medium (BD Co., Sparks, MD, USA) supplemented with 10% horse serum (Gibco, New York, NY, USA). The culture conditions were 37 °C, 100% humidity, and 10% CO_2_ for 2–3 days. The antibacterial activity was evaluated using a previously described method [50]. Briefly, 20 μL of the bacterial colony suspension, equivalent to 2–3 × 10^8^ cfu/mL, was added to the Brucella broth medium supplemented with 10% horse serum in each six-well. The final concentration of the samples and positive control was 100 μM, and the final volume was 2 mL. After 24 h of incubation at 37 °C and 10% CO_2_, the growth was evaluated by measuring the optical density at 600 nm using an Optizen POP UV/VIS spectrophotometer (Mecasys, Daejeon, Korea). The inhibitory activity was obtained based on the following equation:

Inhibition (%) = [(absorbance of the control − absorbance of solution with samples)/absorbance of the control] × 100.

Dimethyl sulfoxide (DMSO) was used as the negative control, while quercetin and an antibiotic in the clinical field, metronidazole, were used as positive controls. Data were expressed as means ± standard deviations (SD) of the double-independent experiments. The statistical differences among the samples were determined by one-way ANOVA using IBM SPSS Statistics 24.0 software (Armonk, NY, USA). The statistical significance level was performed at 5%.

## 4. Conclusions

In this study, we investigated potential bioactive compounds from the ethanol extract of *S. chaenomeloides* leaves. We isolated and identified thirteen compounds, including a new compound, chaenomelin (**1**), from the EtOAc fraction, with antibacterial activity against *H. pylori*. The structure of the new compound was determined using 1D and 2D NMR techniques (^1^H-^1^H COSY, HSQC, and HMBC) and HR-ESIMS. We evaluated all thirteen compounds for their antibacterial activity against *H. pylori* strain 51. Notably, compounds **7** and **8** showed significant antibacterial activity, comparable to quercetin, serving as a positive control. These findings suggest the therapeutic potential of *S. chaenomeloides* and its bioactive compounds **7** and **8**, opening avenues for future research as antibacterial agents against *H. pylori.* Limitations of this study include the need for further investigation into the mechanisms of action of compounds **7** and **8**, as well as their efficacy when in vivo. Additionally, broader screening against a panel of bacterial strains and assessment of cytotoxicity in mammalian cells would provide a more comprehensive understanding of the therapeutic potential and safety profile of these compounds.

## Figures and Tables

**Table 1:** ^1^H (850 MHz) and ^13^C NMR (212.5 MHz) data for compound **1** in CD_3_OD (*δ* ppm) ^a^.

Position	1	
	*δ*_H_ (*J* in Hz)	*δ*_C_
1		127.5 C
2	7.47 d (8.5)	131.3 CH
3	6.83 d (8.5)	116.9 CH
4		161.5 C
5	6.83 d (8.5)	116.9 CH
6	7.47 d (8.5)	131.3 CH
7	7.61 d (16.0)	146.9 CH
8	6.33 d (16.0)	115.4 CH
9		168.6 C
1′	4.56 d (8.0)	101.4 CH
2′	4.89 dd (9.5, 8.0)	75.2 CH
3′	3.56 m	76.2 CH_2_
4′	3.41 m	71.8 CH
5′	3.33 m	78.3 CH
6′	3.93 dd (12.0, 2.0); 3.73 dd (12.0, 6.0)	62.8 CH_2_
1″		139.2 C
2″	7.27 d (7.0)	129.0 CH
3″	7.22 m	129.3 CH
4″	7.22 m	129.1 CH
5″	7.22 m	129.3 CH
6″	7.27 d (7.0)	129.0 CH
7″	4.87 d (12.5); 4.64 d (12.5)	71.6 CH_2_

^a^ Coupling constants (Hz) were given in parentheses, and ^13^C NMR data were assigned based on HSQC and HMBC experiments.

**Table 2:** Anti-*H. pylori* activity of compounds **1–13** against *H. pylori* strain 51 treated with 100 μM of each compound.

Compound	Inhibition (%) ^B^
**1**	21.6 ± 4.4 ^c^
**2**	11.9 ± 0.9 ^d^
**3**	13.6 ± 1.8 ^d^
**4**	19.0 ± 2.8 ^cd^
**5**	16.3 ± 6.3 ^cd^
**6**	13.0 ± 4.4 ^d^
**7**	31.4 ± 3.9 ^b^
**8**	33.9 ± 4.4 ^b^
**9**	1.2 ± 0.7 ^e^
**10**	18.9 ± 0.3 ^cd^
**11**	19.4 ± 1.7 ^cd^
**12**	15.5 ± 0.7 ^cd^
**13**	19.1 ± 3.0 ^cd^
**Quercetin ^A^**	38.4 ± 2.3 ^b^
**Metronidazole ^A^**	96.6 ± 0.5 ^a^

^A^ Positive controls; ^B^ different letters in the same column mean significantly different (*p* < 0.05).

**Table 3:** Equipment used for analyses.

Experimental Procedure	Equipment
Optical rotations	JASCO P-2000 polarimeter (JASCO, Easton, MD, USA)
Ultraviolet (UV) spectra	Agilent 8453 UV-visible spectrophotometer (Agilent Technologies, Santa Clara, CA, USA)
Infrared (IR) spectra	Bruker IFS-66/S FT-IR spectrometer (Bruker, Karlsruhe, Germany)
Nuclear magnetic resonance (NMR) spectra	Bruker AVANCE III HD 850 NMR spectrometer with a 5 mm TCI CryoProbe operating at 850 MHz (^1^H) and 212.5 MHz (^13^C)
HR-ESIMS	Agilent G6545B quadrupole time-of-flight mass spectrometer (Agilent Technologies) Agilent 1290 Infinity II high-performance liquid chromatography (HPLC) instrument (Agilent Eclipse Plus C18 column (2.1 × 50 mm, 1.8 μm; flow rate: 0.3 mL/min))
Preparative HPLC	Waters 1525 Binary HPLC pump with a Waters 996 Photodiode Array Detector (Waters Corporation, Milford, MA, USA) and an Agilent Eclipse C18 column (250 × 21.2 mm, 5 μm; flow rate: 5 mL/min; Agilent Technologies)
Semi-preparative HPLC	Waters 1525 Binary HPLC pump with a Waters 996 Photodiode Array Detector (Waters Corporation, Milford, CT, USA) Phenomenex Luna C18 column (250 × 10 mm, 10 μm; flow rate: 2 mL/min; Phenomenex, Torrance, CA, USA) Phenomenex Luna Phenyl-Hexyl column (250 × 10 mm, 10 μm; flow rate: 2 mL/min; Phenomenex)
LC/MS analysis	Agilent 1200 Series HPLC system equipped with a diode array detector and 6130 Series ESI mass spectrometer using an analytical Kinetex C18 100 Å column (100 × 2.1 mm, 5 μm; flow rate: 0.3 mL/min; Phenomenex).
Column chromatography	Silica gel 60 (230–240 mesh; Merck, Darmstadt, Germany) RP-C18 silica gel (Merck, 230–240 mesh)
Thin-layer chromatography (TLC)	Pre-coated silica gel F254 plates and RP-C18 F254s plates (Merck)

## Data Availability

Data available on request from the corresponding author.

## Supplementary Materials

The following supporting information can be downloaded at: https://www.mdpi.com/article/10.3390/plants13050701/s1, Figure S1: The HR-ESIMS data of compound **1**; Figure S2: The UV spectrum of compound **1**; Figure S3: The ^1^H NMR spectrum of compound **1** (CD_3_OD, 850 MHz); Figure S4: The ^1^H-^1^H COSY spectrum of compound **1**; Figure S5: The HSQC spectrum of compound **1**; Figure S6: The HMBC spectrum of compound **1**.
