# Supplementary material for: Chaenomelin, a New Phenolic Glycoside, and Anti-Helicobacter pylori Phenolic Compounds from the Leaves of Salix chaenomeloides
Source: Plants (Basel). 2024 Feb 29;13(5):701. doi: 10.3390/plants13050701 (PMC10935347; doi:10.3390/plants13050701)
Supplement: Supplementary file 1 [file plants-13-00701-s001.zip › plants-2831314-supplementary.pdf]

# Supplementary Materials

# Chaenomelin, a New Phenolic Glycoside and Anti-*Helicobacter pylori* Phenolic Compounds from the Leaves of *Salix chaenomeloides*

**Kyung Ah Kim [1], Dong-Min Kang [2], Yoon-Joo Ko [3], Moon-Jin Ra [4], Sang-Mi Jung [4], Jeong-Nam Yu [5], Mi-Jeong Ahn [2] and Ki Hyun Kim [1,*]**

1. *School of Pharmacy, Sungkyunkwan University, Suwon 16419, Republic of Korea.*
2. *College of Pharmacy and Research Institute of Pharmaceutical Sciences, Gyeongsang National University, Jinju 52828, Republic of Korea.*
3. *Laboratory of Nuclear Magnetic Resonance, National Center for Inter-University Research Facilities (NCIRF), Seoul National University, Gwanak-gu, Seoul 08826, Republic of Korea*
4. *Hongcheon Institute of Medicinal Herb, Hongcheon-gun 25142, Republic of Korea*
5. *Nakdonggang National Institute of Biological Resources, Sangju 37242, Republic of Kore*

* *Correspondence: E-mail: khkim83@skku.edu; Tel: +82-31-290-7700; Fax: +82-31-290-7730*

**Electronic Supplementary Information Contents:**

**Figure S1**. The HR-ESIMS data of compound **1**……………………………………………………..………………………………….S3

**Figure S2**. The UV spectrum of compound **1**………………………………………………………………………………………….S4

**Figure S3**. The $^{1}$H NMR spectrum of compound **1** ($CD_3OD$, 850 MHz)......…. ..……………………………………………………….S5

**Figure S4**. The $^{1}$H-$^{1}$H COSY spectrum of compound **1**..………………………….……………………….………………………S6

**Figure S5**. The HSQC spectrum of compound **1**………………………………. …………………………….……….……………S7

**Figure S6**. The HMBC spectrum of compound **1**……...………….……………………………………….....………. . ..….…………S8

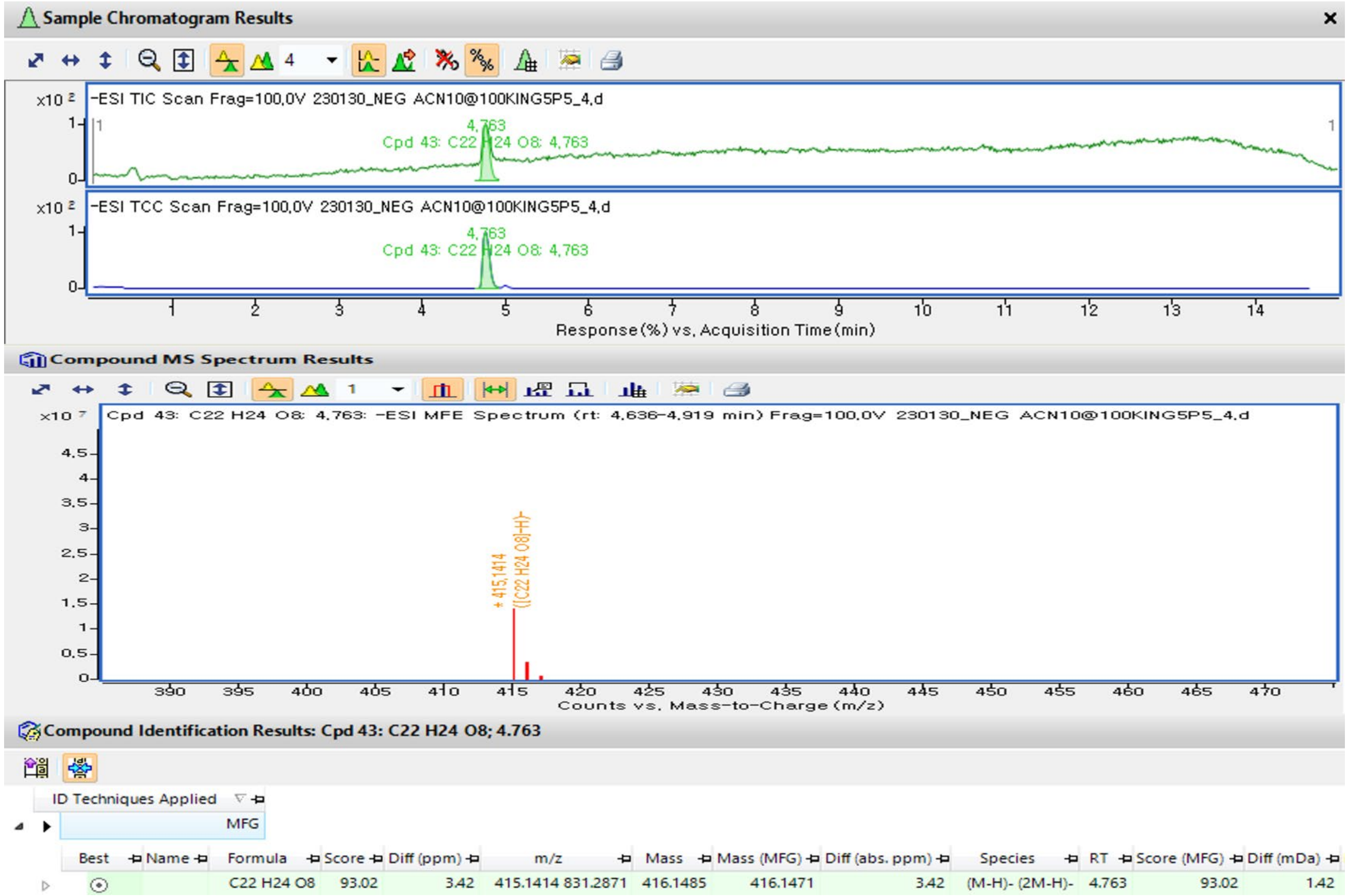


**Figure S1**. The HR-ESIMS data of compound **1.**

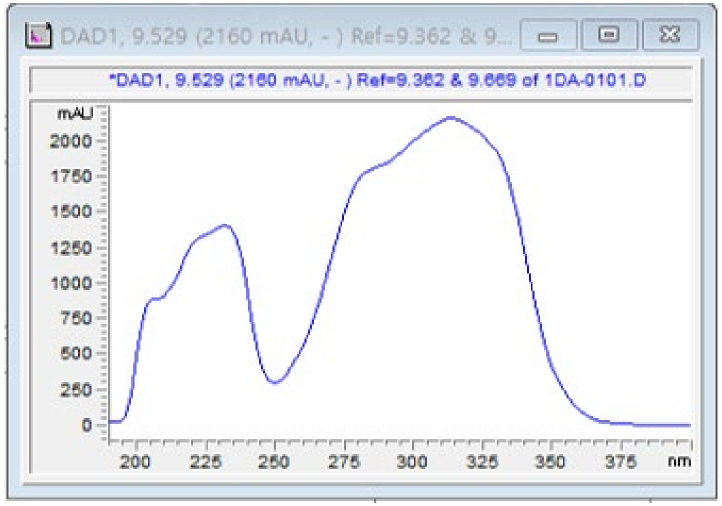


**Figure S2**. The UV spectrum of compound **1.**

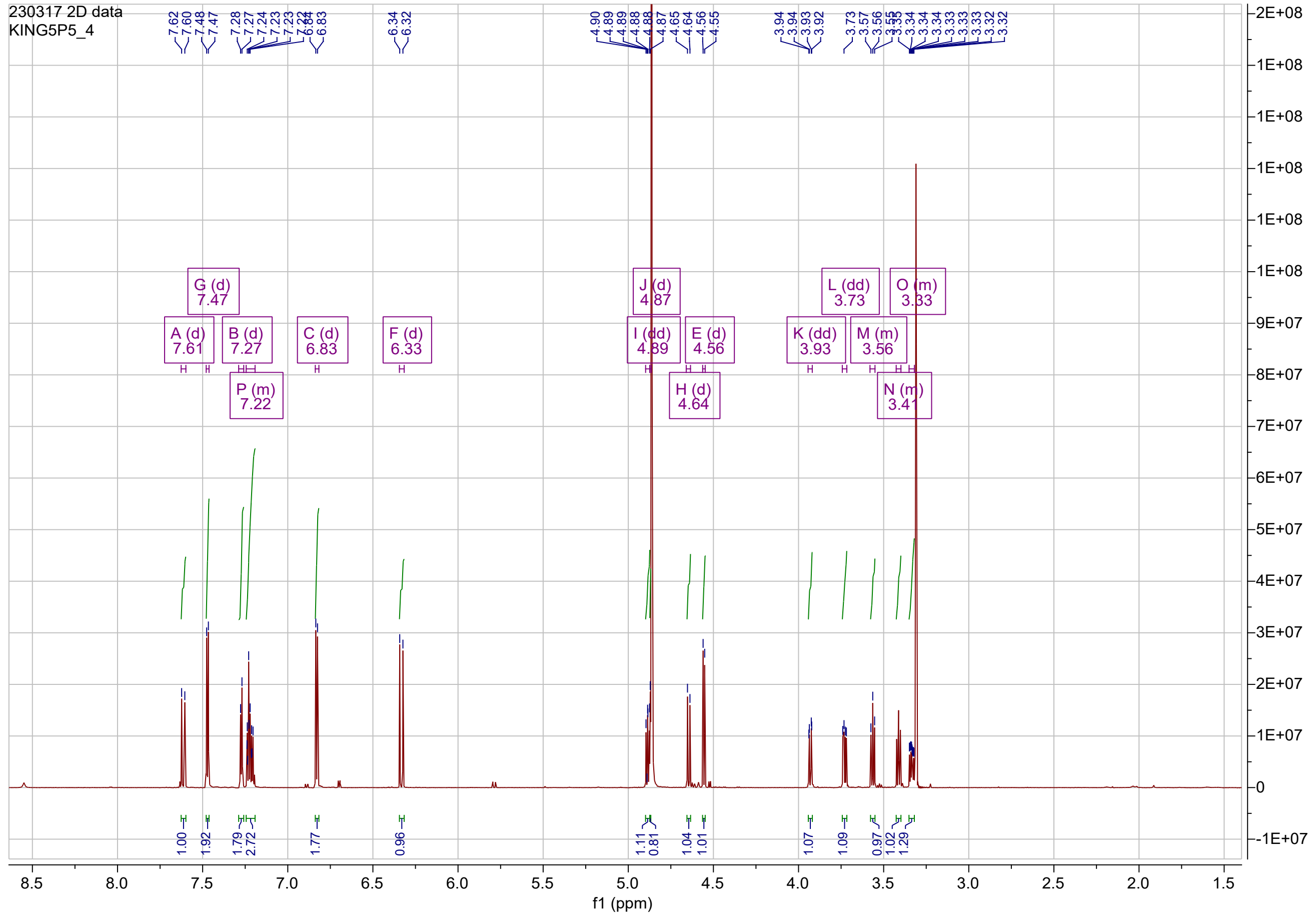

**Figure S3**. The $^{1}H$ NMR spectrum of compound **1** ($CD_3OD$, 850 MHz).

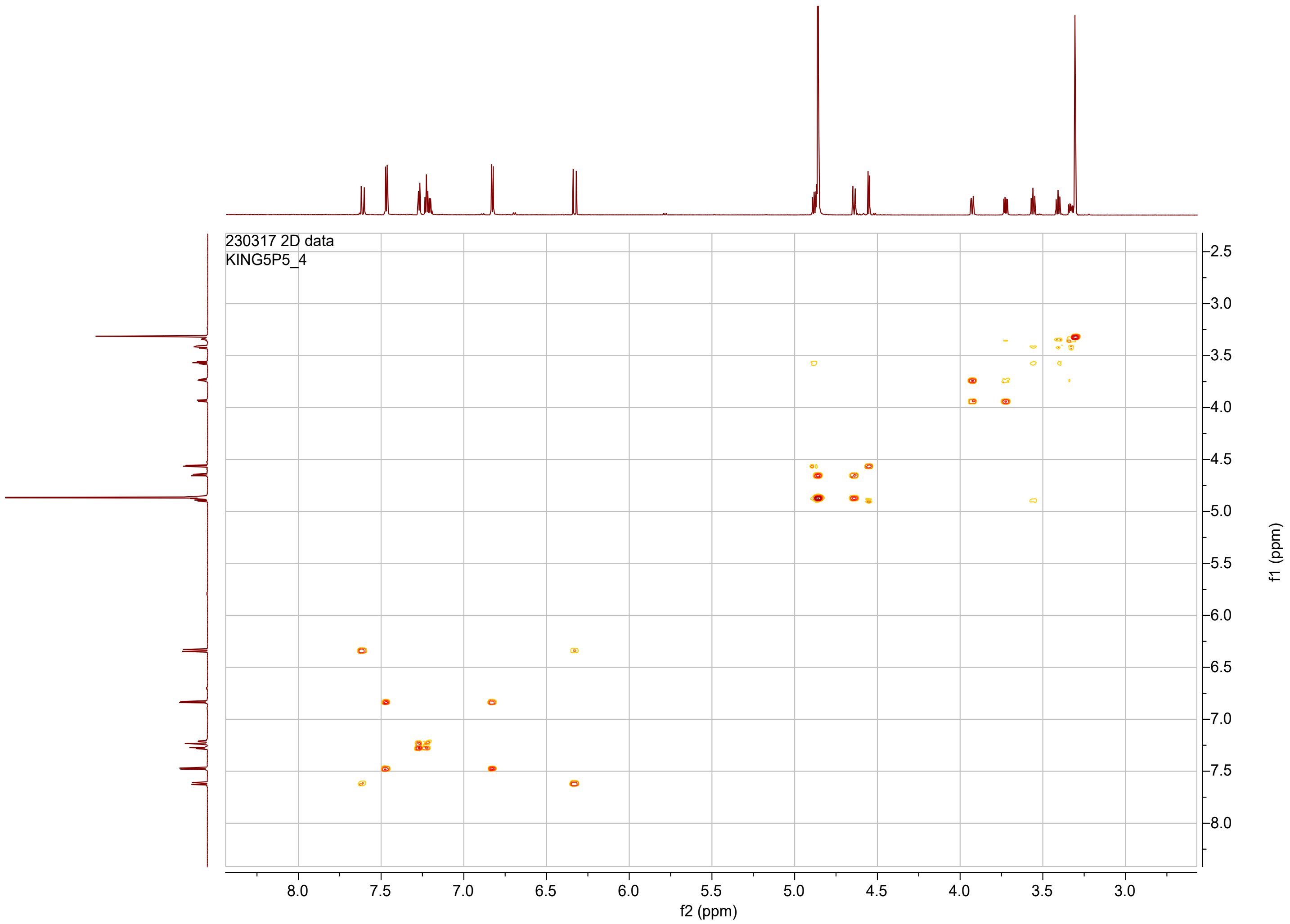


**Figure S4**. The $^{1}$H-$^{1}$H COSY spectrum of compound **1.**

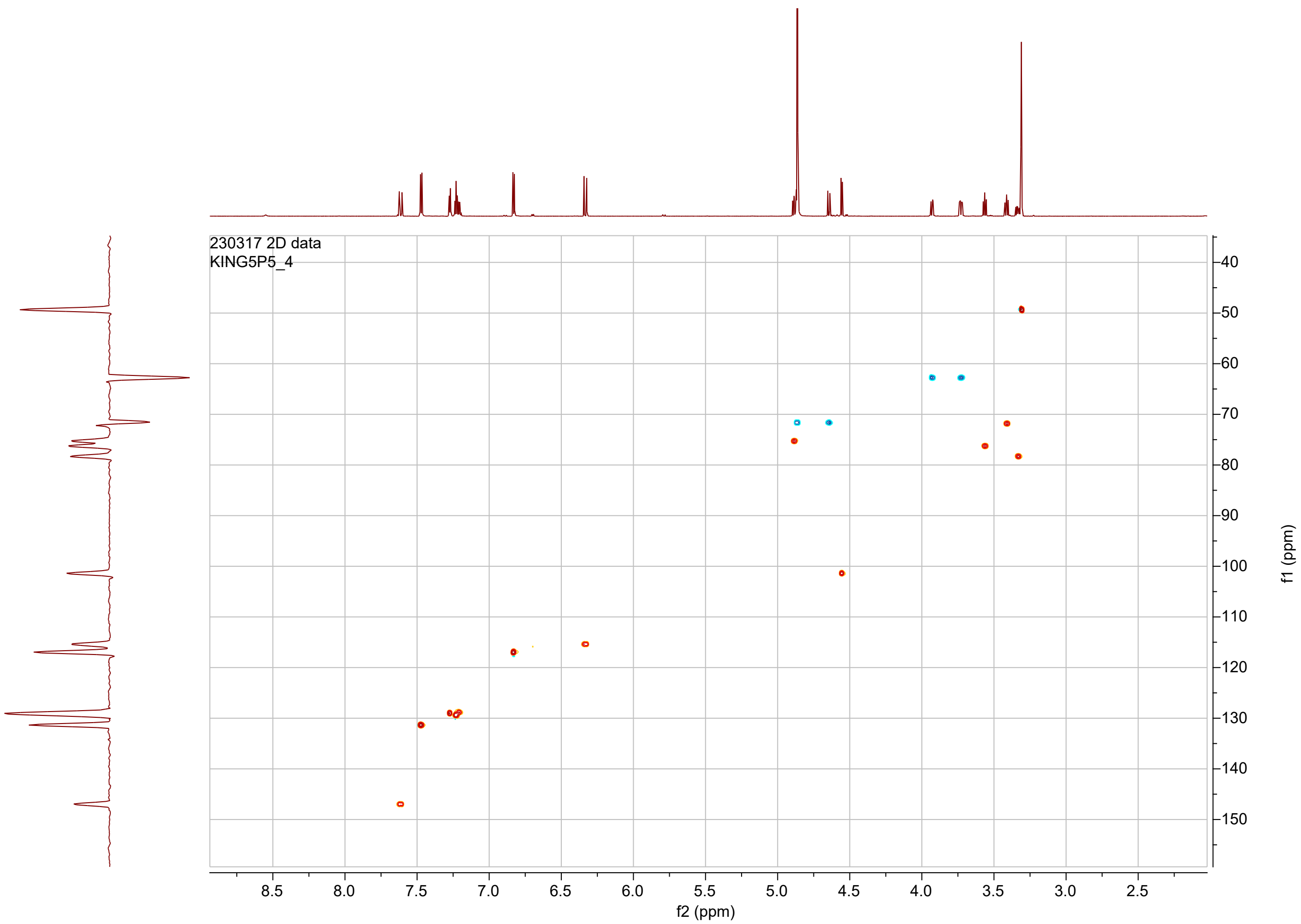


**Figure S5**. The HSQC spectrum of compound **1.**

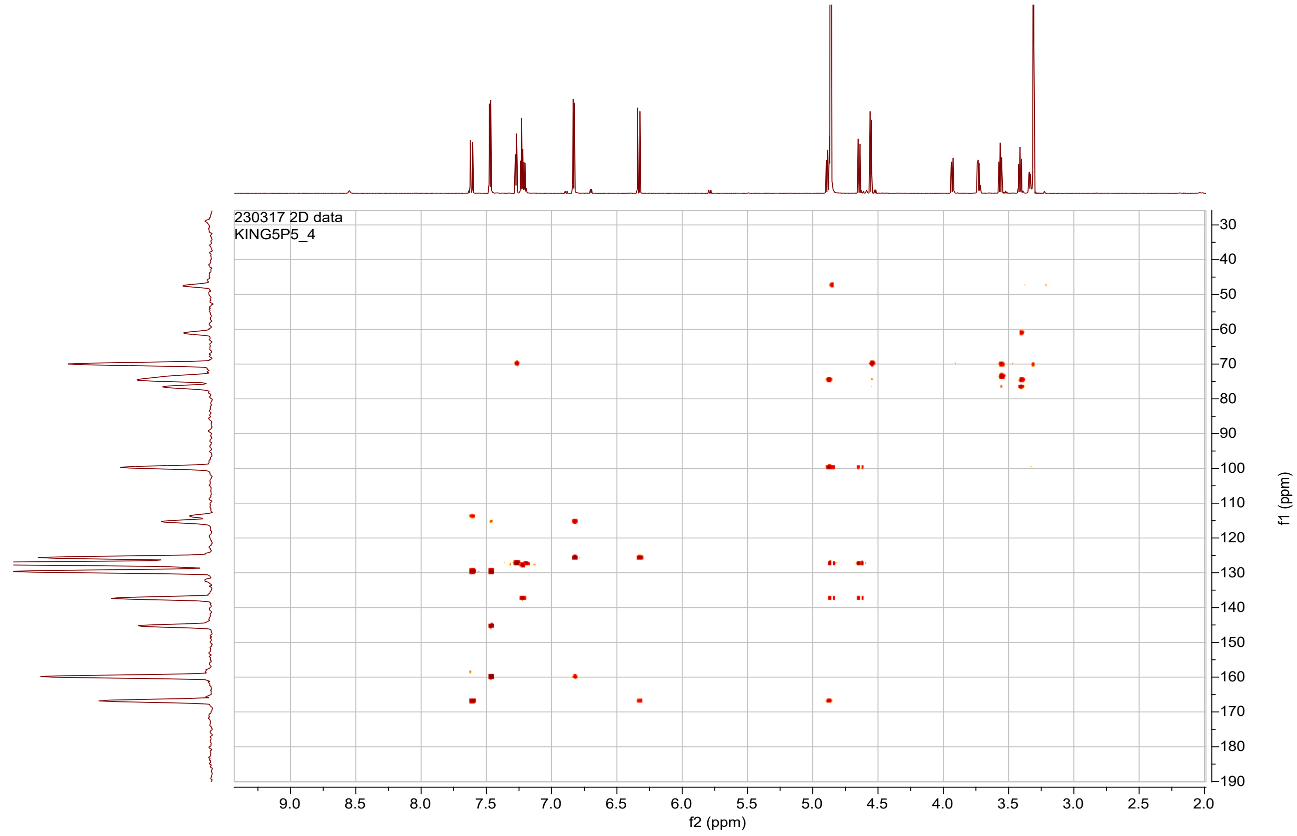


**Figure S6**. The HMBC spectrum of compound **1.**
